# Characterisation of N-terminal pro-brain natriuretic peptide in dialysis patients and its reduced prognostic significance in the elderly

**DOI:** 10.1038/s41598-019-43253-z

**Published:** 2019-04-29

**Authors:** Yuji Sato, Yuri Ishizaki, Kumiko Aso, Akihiro Minakwa, Tatsunori Toida, Ryuzoh Nishizono, Masao Kikuchi, Hiroko Inagaki, Shouichi Fujimoto

**Affiliations:** 1 Dialysis Division, University of Miyazaki Hospital, Miyazaki, Japan; 2 Department of Internal Medicine, Division of Circulatory and Body Fluid Regulation, Faculty of Medicine, University of Miyazaki, Miyazaki, Japan; 3 Department of Hemovascular Medicine and Artificial Organs, Faculty of Medicine, University of Miyazaki, Miyazaki, Japan

**Keywords:** Predictive markers, Renal replacement therapy

## Abstract

Characterisation of N-terminal pro-brain natriuretic peptide (NT-proBNP) in chronic haemodialysis patients and its prognostic significance in age stratification have not been addressed. A prospective cohort study with cross-sectional analyses at baseline was performed. Outcomes were all-cause mortality, non-malignancy-related mortality, and cardiovascular disease (CVD)-related mortality. NT-proBNP was significantly higher in elderly, female, and low dry weight patients. Study patients were divided into two groups: Group-O (≥75 years) and Group-Y (<75 years). The 7-year follow-up receiver operating curve analysis showed that NT-proBNP significantly predicted all outcomes. All-cause mortality cut-off points were significantly different among the groups (total cohort, 5375 pg/mL; Group-Y, 3682 pg/mL; Group-O, 11750 pg/mL). Cox regression analysis showed risks for all outcomes by tertile NT-proBNP significantly higher in the total cohort and Group-Y as adjusted by potential confounders. For all-cause mortality, hazard ratios and 95% confidence intervals (CI) were T2 1.70 (0.89 to 3.25), p = 0.11, T3 2.95 (1.54 to 5.67), p < 0.01 in Group-Y; and T2 1.00 (0.64 to 1.58), p = 1.00; T3 1.50 (0.94 to 2.40), p = 0.09 in Group-O. In conclusion, NT-proBNP was significantly higher in elderly, female, and low dry weight chronic dialysis patients. NT-proBNP was significantly associated with all outcomes. However, this association was reduced in elderly patients.

## Introduction

Brain natriuretic peptide (BNP) and N-terminal pro-brain natriuretic peptide (NT-proBNP) are synthesised and secreted by the heart, especially the ventricles^1^ due to left ventricular stress^2–4^. Levels of BNP and NT-proBNP are a prognostic marker of congestive heart failure (CHF) in non-chronic kidney disease (CKD) patients^5–7^.

The role of natriuretic peptides (NPs) in CKD patients, especially dialysis patients, has been investigated. Among the non-CKD population, natriuretic peptide (NP) levels differ with age, sex^7–9^, and anthropometric parameters, e.g. body mass index (BMI)^10,11^. NP levels have been reported to be higher among elderly and women^7–9^ and lower in people with higher BMI^10,11^. Therefore, the first aim of this study was to investigate whether these findings (as reported in non-CKD population) are the same in a patient population undergoing dialysis.

NT-proBNP was reported to be associated with coronary artery disease^12^, left ventricular dysfunction^13^, and volume overload^14^ as well as associated higher NT-proBNP with predicted worse prognosis, all-cause mortality, and cardiovascular mortality^14,15^. In non-CKD patients, the diagnostic power of NT-proBNP was quite different among elderly vs. non-elderly patients under acute dyspnoeic setting^16^. In addition, elderly patients who were 75 years old or older with CHF did not seem to be able to respond to NP-guided treatment^17^. However, evidence of age-related differences in diagnostic or predictive power for mortality in haemodialysis patients is lacking. Therefore, the second aim of this study was to analyse the age-related difference in NT-proBNP predictability for mortality in chronic haemodialysis patients.

## Results

### Participant enrolment

**Figure 1:**
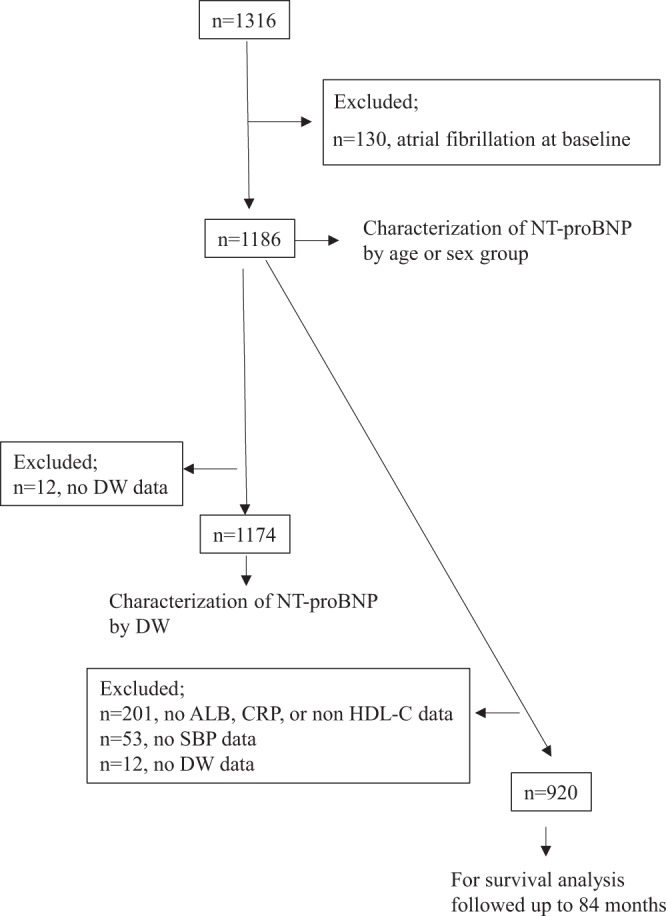
Patient enrolment. Three cohort sets were available: 1186 patients for age or sex group analysis without atrial fibrillation, 1174 patients for anthropometric analysis based on DW, and 920 patients for survival analysis. ALB, albumin; CRP, C-reactive protein; DW, dry weight; HDL-C, high-density lipoprotein cholesterol; SBP, systolic blood pressure

A total of 1316 participants were considered for this study from which 130 patients were excluded due to baseline atrial fibrillation. Finally, 1186 patients were included in the analysis of NT-proBNP characterisation by sex and age group. To investigate the association of anthropometric factors (i.e. dry-weight, DW) to NT-proBNP, 1174 patients (12 patients were excluded as DW data were not available) were included in the association analysis of NT-proBNP by DW. A total of 920 patients [266 patients were excluded as laboratory data (albumin (Alb), C-reactive protein (CRP), and non-HDL-c (non-high-density lipoprotein cholesterol)), systolic blood pressure (SBP), or DW data were not available] were followed up to 84 months and were included in the survival analysis (Fig. 1). To include as many patients as possible, three cohorts (n = 1186, n = 1174, and n = 920) as shown in Fig. 1 were used. The first two cohorts were available for cross-sectional studies of sex and age differences and dry weight difference studies. The last cohort was intended for survival analysis because all- confounder values were available. All three cohorts were not significantly different in basic clinical parameters (S-Table 1).

### Cross-sectional studies at baseline

**Figure 2:**
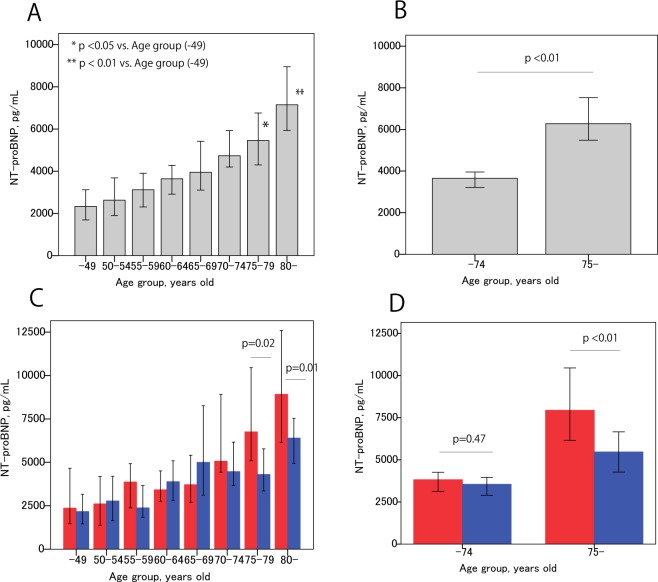
NT-proBNP association with age and sex. (**A,B**) NT-proBNP values and their association with age. Data are expressed as median +/− 95% confidence interval. As shown in (**A**). NT-proBNP increases with age linearly; however, we separated the patient population into two groups (at age 75). Older patients had significantly higher NT-proBNP than younger patients. (**C**,**D**) Relationship between age and sex with NT-proBNP. Sex was more closely associated in the elderly patient population (**C**) (red column, women; Blue column, men), while no sex association was observed in younger patients. Higher NT-proBNP was observed in women than in men among older patients (**D**). P-values were checked by nonparametric test.

NT-proBNP levels in patients aged 75 or older was significantly higher than in patients aged 49 or younger (Fig. 2A). Older patients (75 years old or older, Group-O) had significantly higher NT-proBNP values than younger patients (74 years old and younger, Group-Y); 6275 (1285–67752) vs. 3650 (782–34885), p < 0.01, median (5–95 percentile) (Fig. 2B). The effect of sex on NT-proBNP was more prominent in Group-O than in Group-Y with significantly higher NT-proBNP values among women than men in Group-O (Fig. 2C,D). Figure 2A–D show the median +/− 95% CI of NT-proBNP.

In addition to age and sex, we investigated the association of an anthropometric factor (DW) with NT-proBNP. Raw DW data were divided into quartiles, and the standard DW score was calculated for each sex. Data for each sex was compiled and divided into quartiles. NT-proBNP in quartiles 2, 3, and 4 were significantly lower than that in quartile 1 for raw data (S-Fig. 1A) and standard score (S-Fig. 1B).

Participants were divided into two age groups (Group-Y and Group-O), and each group was further divided into tertiles of log NT-proBNP. Participant characteristics are shown in Table 1. Raw NT-proBNP value and log NT-proBNP were different among tertiles in the two age groups. Other clinical information and laboratory data (such as age, sex, DW standard score, cardiothoracic ratio (CTR), Alb, CRP, etc.) were also significantly different among the tertiles. Diabetes as a basal kidney disease, per cent of inter-dialytic body weight gain compared to dry weight, mean predialysis SBP, anti-hypertensive drug use, and non-HDL cholesterol were significantly different among tertiles in Group-Y but not in Group-O.

**Table 1:** Patients’ characteristics according to two age groups and log NT-proBNP tertile

Tertile of Log NT-proBNP	Group-Y (age < 75)				Group-O (age ≧ 75)			
	Tertile 1	Tertile 2	Tertile 3	p-value	Tertile 1	Tertile 2	Tertile 3	p-value
Number	204	203	204		103	103	103	
NT-proBNP, pg/mL	1385 (570–2127)	3650 (2337–5536)	11953 (6247–86425)	<0.01	2690 (852–4042)	6080 (4274–10124)	24100 (11311–130400)	<0.01
Log NT-proBNP, pg/mL	3.10 (0.18)	3.56 (0.12)	4.17 (0.34)	<0.01	3.38 (0.21)	3.80 (0.12)	4.42 (0.31)	<0.01
Age, years	58.2 (10.3)	62.4 (8.9)	62.5 (8.5)	<0.01	80.3 (4.6)	81.4 (4.9)	82.1 (5.8)	0.04
Sex, women, %	36.3%	48.8%	37.3%	0.02	37.9%	51.5%	57.3%	0.02
**Dry weight, kg**								
Women	51.9 (10.4)	48.1 (8.2)	46.3 (9.5)	<0.01	44.8 (9.1)	44.6 (7.3)	41.3 (7.7)	0.04
Men	62.0 (10.6)	58.9 (10.1)	57.3 (9.8)	<0.01	55.3 (8.1)	53.6 (7.5)	50.2 (6.1)	<0.01
Standard score of dry weight	54.8 (10.6)	51.4 (9.3)	49.7 (9.8)	<0.01	47.9 (8.6)	46.9 (7.6)	43.6 (7.3)	<0.01
Log dialysis vintage, months	1.90 (0.38)	2.01 (0.35)	1.99 (0.33)	0.01	1.71 (0.33)	1.87 (0.33)	1.88 (0.32)	<0.01
Cardiothoracic ratio, %	48.1 (4.2)	50.2 (4.1)	53.3 (5.5)	<0.01	51.1 (4.8)	52.8 (4.1)	55.8 (5.4)	<0.01
Basal kidney disease, diabetes, %	19.6%	18.7%	35.3%	<0.01	21.4%	17.5%	22.3%	0.66
Inter-dialytic body weight gain, %	4.17 (1.55)	4.76 (1.57)	5.11 (2.19)	<0.01	4.34 (1.67)	4.70 (1.70)	4.58 (2.15)	0.40
CVD history, %	21.6%	23.2%	38.2%	<0.01	31.1%	37.9%	51.5%	0.01
Current smoking status, %	17.6%	15.3%	22.1%	0.20	8.7%	7.8%	6.8%	0.87
Predialysis SBP, mmHg	149 (19)	157 (18)	160 (19)	<0.01	155 (16)	155 (18)	156 (19)	0.94
Antihypertensive drug use, %	68.6%	78.3%	83.8%	<0.01	73.8%	78.6%	77.7%	0.68
Albumin, g/dL	4.06 (0.40)	3.97 (0.41)	3.87 (0.54)	<0.01	3.68 (0.36)	3.62 (0.43)	3.52 (0.45)	0.02
Log C-reactive protein, mg/dL	−0.95 (0.52)	−0.97 (0.50)	−0.73 (0.64)	<0.01	−0.80 (0.54)	−0.77 (0.59)	−0.59 (0.58)	0.02
Non-HDL cholesterol, mg/dL	115 (29)	116 (33)	109 (33)	0.03	115 (27)	111 (31)	109 (30)	0.28

CVD, cardiovascular disease; HDL, high-density lipoprotein; NT-proBNP, N-terminal pro-brain natriuretic peptide; SBP, systolic blood pressure.

NT-proBNP, median (25–75 percentile).

The per cent of the inter-dialytic body weight gain was calculated as mean 3-consecutive inter-dialytic body weight gain (kg) comparable to dry weight (kg).

### Survival analysis

During follow-up (up to 84 months), a total of 306 patients died (274 non-malignancy-related deaths and 127 CVD-related deaths) (S-Table 2), and the main causes of death are shown in S-Table 3.

Kaplan-Meier survival estimate curves showed that tertiles of log NT-pro BNP were significantly and clearly differentiated for all-cause mortality in Group-Y (S-Fig. 2A) and Group-O (S-Fig. 2B), non-malignancy-related mortality in Group-Y (S-Fig. 2C) and Group-O (S-Fig. 2D), and CVD-related mortality (S-Fig. 2E,F).

**Figure 3:**
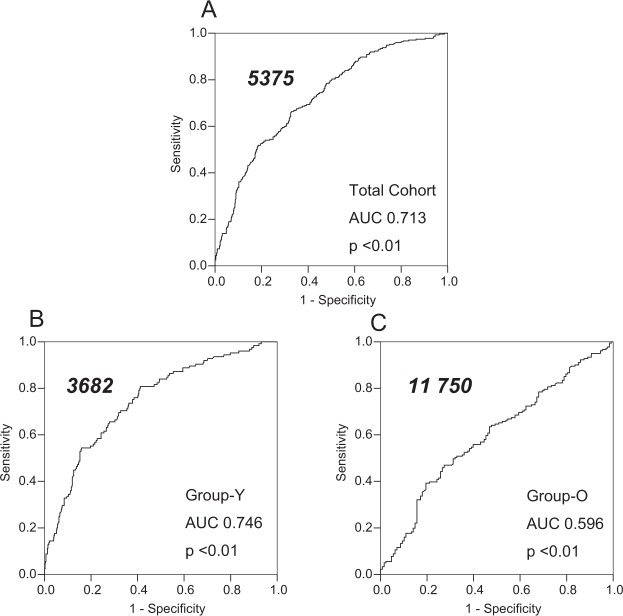
Receiver operating characteristic curves of log NT-proBNP for all-cause mortality. Receiver operating characteristic curves of log NT-proBNP for all-cause mortality are shown in different cohorts: total cohort (**A**), Group-Y (age < 75 years) (**B**), and Group-O (age ≥ 75 years) (**C**). Area under the curve (AUC) and p-value for each cohort are shown. The AUC of the total cohort and Group-Y had a modest predicting value; however, the AUC of Group-O was not predictive. Cut-off point of each cohort is shown in each figure as bold italic number and was remarkably different between Group-Y and Group-O. Therefore, same NT-proBNP value may not have the same significance for younger vs. older dialysis patients

Receiver operating characteristic (ROC) curves of NT-proBNP among the total cohort (Fig. 3A), Group-Y (Fig. 3B), and Group-O (Fig. 3C) for all-cause mortality are presented. AUC and 95% CI for the total cohort, Group-Y, and Group-O were 0.713 (0.678 to 0.747), p < 0.01; 0.746 (0.698 to 0.795), p < 0.01; and 0.596 (0.532 to 0.660), p < 0.01, respectively. Cutoff values of NT-proBNP for all-cause mortality for the total cohort, Group-Y, and Group-O were 5375 pg/mL, 3682 pg/mL, and 11750 pg/mL, respectively. ROC analyses for non-malignancy- and CVD-related mortality were similar to all-cause mortality.

Cox regression analysis showed that tertile 3 and/or tertile 2 of log NT-proBNP had significantly higher hazard ratios (HRs) than tertile 1 for all-cause mortality, non-malignancy-related mortality, and CVD-related mortality in a non-adjusted model (Table 2). Although log NT-proBNP was significantly associated with all outcomes for Group-Y in the adjusted model, the log NT-proBNP did not show a significant association with all outcomes in Group-O. The risks in tertile 3 of log NT-proBNP for all-cause mortality (adjusted model) were 2.95 (1.54 to 5.67), p < 0.01, and 1.50 (0.94 to 2.40), p = 0.09 for Group-Y and Group-O, respectively. HRs and 95% CIs of confounders (adjusted model) are shown in S-Table 4. Alb and history of CVD had strong associations with death in Group-O but not in Group Y. Cox regression analysis including interaction terms for age and log NT-proBNP showed that there was a significant trend for HR for all-cause mortality according to the interaction terms in the unadjusted model (p for interaction = 0.01), but not significant in the fully adjusted model (p for interaction = 0.33) (S-Table 5). We considered the effect modification by age was possible, although it was not officially significant.

**Table 2:** Risks of tertiles log NT-proBNP for mortalities up to 7-year follow-up.

			All-cause death		Non-malignancy-related death		CVD-related death	
			HR (95% CI)	p-value	HR (95% CI)	p-value	HR (95% CI)	p-value
Unadjusted	Total cohort	T1	ref		ref		ref	
		T2	2.35 (1.66 to 3.34)	<0.01	2.51 (1.72 to 3.67)	<0.01	2.68 (1.52 to 4.72)	<0.01
		T3	5.08 (3.67 to 7.03)	<0.01	5.61 (3.94 to 7.99)	<0.01	6.06 (3.56 to 10.31)	<0.01
	Group-Y	T1	ref		ref		ref	
		T2	2.60 (1.40 to 4.85)	<0.01	3.33 (1.57 to 7.06)	<0.01	2.84 (1.11 to 7.27)	0.03
		T3	6.99 (3.95 to 12.36)	<0.01	9.71 (4.84 to 19.45)	<0.01	8.37 (3.55 to 19.74)	<0.01
	Group-O	T1	ref		ref		ref	
		T2	1.21 (0.83 to 1.76)	0.32	1.24 (0.83 to 1.84)	0.29	1.71 (0.90 to 3.24)	0.1
		T3	2.17 (1.52 to 3.09)	<0.01	2.38 (1.64 to 3.44)	<0.01	2.65 (1.42 to 4.96)	<0.01
Adjusted	Total cohort	T1	ref		ref		ref	
		T2	1.49 (1.01 to 2.20)	0.04	1.50 (0.99 to 2.26)	0.06	1.73 (0.93 to 3.19)	0.08
		T3	2.36 (1.59 to 3.51)	<0.01	2.35 (1.54 to 3.59)	<0.01	2.93 (1.57 to 5.46)	<0.01
	Group-Y	T1	ref		ref		ref	
		T2	1.70 (0.89 to 3.25)	0.11	2.07 (0.95 to 4.51)	0.07	1.91 (0.72 to 5.06)	0.20
		T3	2.95 (1.54 to 5.67)	<0.01	3.78 (1.74 to 8.22)	<0.01	3.66 (1.39 to 9.65)	0.01
	Group-O	T1	ref		ref		ref	
		T2	1.00 (0.64 to 1.58)	1.00	0.98 (0.61 to 1.55)	0.92	1.51 (0.72 to 3.16)	0.27
		T3	1.50 (0.94 to 2.40)	0.09	1.49 (0.92 to 2.41)	0.10	1.88 (0.85 to 4.17)	0.12

Adjusted model. Adjusted with age (+1 SD), DW standard score (+1 SD), sex, Alb (+1 SD), log CRP, CVD history, SBP (+1 SD), CTR (+1 SD), non-HDL-c (+1 SD), log dialysis vintage, current smoking, inter-dialytic body weight gain comparable to dry weight (+1%), basal kidney disease (diabetes), and antihypertensive medicine use.

To alleviate the potential concern that a long follow-up (up to 7 years) period in elderly patients may reduce the significance of NT-proBNP’s association with mortality, we evaluated the NT-proBNP for a short-term follow-up duration (3.5 years) in the same cohort (S-Table 6). Similar to the long-term follow-up analysis, the risks of tertile NT-proBNP for each outcome were not significant or reduced in Group-O. The risks for each type of mortality in Group-Y were not calculated because of the small number of recorded death in Group-Y (S-Table 2).

The discriminative ability of the tertile of log NT-proBNP for each mortality was evaluated. The C-index for each endpoint was calculated for the base model and the base model plus tertiles of log NT-proBNP. The addition of tertiles for log NT-proBNP resulted in a significant increase in the C-index for non-malignancy-related mortality and CVD-related mortality in Group-Y, but not in Group-O. The C-index of Group-Y for all-cause mortality was higher than that of Group-O, but did not show statistical significance (Table 3).

**Table 3:** Harrell’s C statistics for Cox regression models predicting each outcome.

		Model	Index	P-value vs. base model
All-cause mortality	Group-Y	Base model	0.69 (0.63 to 0.76)	0.11
		Base model + tertile log NT-proBNP	0.73 (0.67 to 0.79)	
	Group-O	Base model	0.69 (0.62 to 0.75)	1.00
		Base model + tertile log NT-proBNP	0.69 (0.63 to 0.75)	
Non-malignancy-related mortality	Group-Y	Base model	0.67 (0.60 to 0.74)	<0.01
		Base model + tertile log NT-proBNP	0.73 (0.67 to 0.79)	
	Group-O	Base model	0.67 (0.61 to 0.74)	0.82
		Base model + tertile log NT-proBNP	0.67 (0.61 to 0.74)	
CVD-related mortality	Group-Y	Base model	0.62 (0.53 to 0.71)	<0.01
		Base model + tertile log NT-proBNP	0.72 (0.64 to 0.80)	
	Group-O	Base model	0.68 (0.56 to 0.80)	0.89
		Base model + tertile log NT-proBNP	0.68 (0.57 to 0.79)	

Base model consists of age (+1 SD), sex, DW standard score (+1 SD), log CRP, and serum albumin (+1 SD).

## Discussion

This study has three main findings. First, the association of age, sex, and body weight with NT-proBNP values in dialysis patients was similar to that in the non-CKD population^8–11^ despite the difference in absolute values between the these groups. Second, NT-proBNP was significantly associated with all-cause mortality, non-malignancy-related mortality, and CVD-related mortality based on long-term follow-up (7 years) of dialysis patients. Third, the significance of NT-proBNP in predicting death was reduced or absent in elderly dialysis patients (75 years or older) based on both long-term (7 years) and short-term (3.5 years) follow-up.

The reason for the higher NT-proBNP values in elderly patients and their lack of significance in predicting death could not be conclusively determined. BNP and NT-proBNP are generated in the same molar quantities from proBNP and are released into the blood. BNP is metabolised by natriuretic receptor-A (NPR-A) and NPR-C of adipose tissue, neutral endopeptidase, and kidney. NT-proBNP is metabolised by the kidney^18–20^. Even though the two NPs are metabolised differently, their associations with age, sex, kidney function, and BMI are similar^7–11^. Therefore, the difference in their behaviour can primarily be explained by the differences in their synthesis and/or secretion from the heart. We attempted to investigate the reasons for the difference in NT-proBNP values at baseline and the reduction of their prognostic significance. Skeletal muscle mass loss, malnutrition, protein energy wasting, volume overload, left ventricular dysfunction, and sympathetic nerve enhancement were candidate explanatory factors.

First, NP levels may be related to hydration status and muscle mass. Studies have reported that elderly people with or without comorbidities have increased extracellular water (ECW). It has been indicated that muscle mass loss primarily causes increased ECW because muscles consist of 70%-80% water—mainly intracellular water (ICW)^21^. ECW is reportedly higher in elderly subjects than in healthy adults; moreover, a higher proportion of total body water (TBW) is composed of ECW in elderly patients^22^ as well as those with CHF^23^. Silva *et al*. reported that older subjects had smaller lean soft tissue mass (the skeletal mass is the main component) than their younger counterparts^24^. They also revealed that aging was associated with an absolute ECW increase, absolute ICW decrease, and an increase in the ECW/ICW ratio. This latter finding and pattern was consistent with previous studies^25^. Skrabal *et al*.^26^ reported interesting findings that increase in fat mass is almost equal to the decrease in muscle mass^24^, resulting in an increase in ECF/ICF ratio, and this ratio was further increased in patients with CHF or undergoing dialysis. Based on these findings, chronic volume overload status due to decreasing skeletal muscle mass may stimulate synthesis and secretion of NPs.

Second, the relationships between NP value and muscle mass, malnutrition, sympathetic nerve activity, or protein energy wasting (PEW) were reported. Das *et al*. reported that both BNP and NT-proBNP (NPs) were more closely associated with lean mass than fat mass in a Dallas heart patient population^27^. Booth *et al*. reported that NT-proBNP level was mainly influenced by hydration status rather than cardiac function and that the hydration status could be enhanced by malnutrition in dialysis patients^28^. Kamada *et al*. recently showed a significant negative correlation between skeletal muscle strength and BNP or left ventricular mass index (LVMI) in haemodialysis patients^29^. They proposed that potentially activated sympathetic nerve system due to weak muscle strength facilitated LVMI increase. A recent study of prevalent dialysis patients^30^ reported that NT-proBNP could be a biomarker of PEW. Moreover, NT-proBNP was reported higher in dialysis patients with increased levels of hsCRP and interleukin-6 and serially decreased index of muscle mass. These findings append some accessory pathways from skeletal muscle mass loss and higher NP values.

Third, the remote relationship between low cardiac function and higher NP values was shown by Booth^28^. Further, Fagugli *et al*. reported higher BNP values in volume-overload haemodialysis patients without cardiac dysfunction^31^.

In elderly dialysis patients, loss of muscle mass could increase ECF, which chronically stimulates NP synthesis and secretion. In other words, a cachexic patient can easily suffer from volume overload. This can possibly explain why an elderly, female, or low BMI patient has higher NPs due to low muscle mass. Other mechanisms such as increased sympathetic nerve system may be also involved. However, this concept is speculative because we had no data regarding muscle mass, nutritional status, PEW, etc. Therefore, future studies should focus on the relationship between NPs and these factors.

There may be additional important risk factors that associate with or predict mortality in elderly patients. Albumin may be a factor, as it was not significant in younger cohort, but was significant in elderly patients based on the Cox regression analysis. Moreover, unknown confounders were possibly not fully adjusted in the Cox regression analysis. Therefore, NT-proBNP was not determined to be a significant predictor of mortality in the elderly cohort.

Association between age and NT-proBNP as risk factor for mortality should be linear; however, we included a breakpoint at age 75 and demonstrated that the significance of NT-proBNP as a risk factor for mortality was reduced or absent in patients aged 75 years or older. Age 75 years was determined as the cut-off age, as NT-proBNP levels of elderly patients (≥75 years) were significantly higher than those of younger patients (<50 years), and age 75 years is the cut-off age for differential diagnosis of acute heart failure from respiratory failure in a setting of acute dyspnoeic patients^16^. An age-adjusted cut-off point strategy was previously shown to be far superior to a single diagnostic cut-off point. Furthermore, NP-guided treatment for chronic heart failure did not seem to be effective in patients aged 75 years or older^17^. Most studies set a single target level of NP. This strategy was thought to be unreasonable because of the large age difference associated with NPs.

This study has some limitations. First, the predicting value of NT-proBNP for mortality should be linear with age; however, we only divided the patient population into two age groups separated at age 75. We could have divided into more age groups of 5 years or 10 years intervals; however, we have not performed this because of the limited number of study participants for each age group. Second, the anthropometric difference analysis in this study was insufficient. BMI is used widely when comparing anthropometric difference; however, our dataset did not include body height. Therefore, only DW was available for analysis of anthropometric differences. Third, as we proposed the importance of muscle mass loss, malnutrition, or PEW, we should have included an index regarding muscle mass, such as creatinine index (a significant risk factor for mortality in dialysis patients)^32^; however, our data set did not include serum creatinine levels. Fourth, our cohort is a prevalent cohort; therefore, we cannot deny that patients who were highly susceptible to death were already dead before the study enrolment.

In conclusion, NT-proBNP values were higher in elderly, female, and low DW chronic prevalent dialysis patients. The prognostic significance for mortality was reduced in the elderly patients. Similar NT-proBNP values do not indicate the same level of significance between older and younger patients. Therefore, we believe that physicians must carefully consider NT-proBNP values, especially in elderly dialysis patients.

## Methods

### Study design and participants

A prospective cohort study with cross-sectional analyses at baseline was performed. The study included 1316 patients with prevalent chronic haemodialysis maintained on outpatient dialysis at 27 dialysis centres enrolled on December 31, 2010, and followed up to 7 years. The exclusion criteria included less than 3 months of haemodialysis, patients under 18 years old, pregnant women, hospitalised patients, and patients who did not provide consent for the study.

### Data collection

Clinical data and survival information (death and cause of death) was collected from questionnaires as documented by the attending physicians. The mean pre-dialysis blood pressure and mean inter-dialytic body weight gain were calculated using data from three consecutive dialysis sessions. Laboratory data of Alb, CRP, non-HDL cholesterol (total cholesterol minus HDL-c), as well as NT-proBNP were measured using the samples drawn from pre-dialysis timing of the first dialysis session of the week (Monday or Tuesday). Chest X-ray was also taken in the pre-dialysis session of the first dialysis session of the week (Monday or Tuesday). NT-proBNP was measured using ECLIA method at SRL Inc. (Tokyo). As the raw DW value was remarkably different between women and men, the standard score of DW was calculated for each sex and compiled. Standard score was calculated as follows:

$${\rm{Standard}}\,{\rm{score}}=50+10({\rm{score}}\mbox{--}{\rm{mean}})/{\rm{standard}}\,{\rm{deviation}}$$

Past CVD history included stroke, ischaemic heart disease (myocardial infarction, angina, coronary artery bypass grafting, and percutaneous transluminal coronary angioplasty), CHF, and peripheral artery diseases. CVD-related death was defined as death from stroke, ischaemic heart disease, CHF, sudden death, and aortic dissection or rupture.

### Ethical consideration

This study was performed in accordance with the principles of the Declaration of Helsinki and was approved by the University of Miyazaki Research Ethics Committee (No. 740). This was a non-invasive observational study, and all information was anonymised. Verbal informed consent was taken and recorded on patient medical charts by attending physicians. A poster announcing the study and stating that all participants had the right to reject participation in the study was placed in a conspicuous location at each dialysis clinic or centre.

### Statistical analysis

Data were expressed as median +/− 95% CI, or mean ± standard deviation (SD) as appropriate. Values of NT-proBNP based on age, sex, or DW were compared using a non-parametric test as appropriate (Mann–Whitney U test or Kruskal–Wallis test). Clinical parameters based on the classification of log NT-proBNP tertiles and age divided at 75 years were compared using one-way analysis of variance or the Kruskal–Wallis test (as appropriate). Categorical parameters were compared using the chi-squared test.

The survival curves according to log NT-proBNP tertiles were analysed using the Kaplan–Meier method, and statistical significance was determined using the log-rank test. The diagnostic performance of log NT-proBNP for mortality was assessed by analysis of the ROC curve. The ROC curve was a plot of sensitivity vs. (1-specificity) for all possible cutoff values. The Youden index method was used to identify the optimal cutoff point^33^.

Cox regression analysis was used to examine and compare log NT-proBNP tertiles associated with mortality. HRs and 95% CIs for all-cause mortality, non-malignancy-related mortality, and CVD-related mortality were independently determined using unadjusted and adjusted models. Adjustment included age (+1 SD), DW standard score (+1 SD), sex, serum Alb (+1 SD), log CRP, past CVD history, pre-dialysis SBP (+1 SD), CTR (+1 SD), non-HDL-c (+1 SD), log dialysis vintage, inter-dialytic body weight gain, basal kidney disease (diabetes), current smoking status, and antihypertensive medication use. The interaction of age on the relationship between NT-proBNP and all-cause mortality was assessed by Cox regression model including age, log NT-proBNP, and the interaction terms for age and log NT-proBNP.

After application of the base model [age (+1 SD), DW standard score (+1 SD), sex, serum Alb (+1 SD), and log CRP] to each endpoint, addition of tertile of log NT-proBNP discrimination was tested using Harrell’s C-index. Selection criteria, except age and sex for C-statistics, had p-values less than 0.01 in the Cox analysis using total cohort for all-cause mortality. Analyses were performed using SPSS version 20.0 J software (IBM Corp., Armonk, NY, USA), except for Harrell’s C-index, for which the differences between the models were analysed using STATA/MP version 14 software (StataCorp) as described in a previous report^34^.

## Supplementary information

Dataset 1

## Data Availability

The dataset analysed in this study are not available due to ethical reasons.
