# Supplementary material for: Characterisation of N-terminal pro-brain natriuretic peptide in dialysis patients and its reduced prognostic significance in the elderly
Source: Sci Rep. 2019 Apr 29;9:6630. doi: 10.1038/s41598-019-43253-z (PMC6488624; doi:10.1038/s41598-019-43253-z)
Supplement: Supplementary file 1 — Dataset 1 [file 41598_2019_43253_MOESM1_ESM.docx]

**Supplementary Information**

**Characterization of N-terminal pro-brain natriuretic peptide in dialysis patients and its reduced prognostic significance in the elderly**

Yuji Sato^1^*, Yuri Ishizaki^2^, Kumiko Asoh^2^, Akihiro Minakwa^2^, Tatsunori Toida^3^, Ryuzoh Nishizono^2^, Masao Kikuchi^2^, Hiroko Inagaki^1^, Shouichi Fujimoto^3^

^1^Dialysis Division, University of Miyazaki Hospital, Miyazaki, Japan

^2^ Department of Internal Medicine, Division of Circulatory and Body Fluid Regulation, Faculty of Medicine, University of Miyazaki, Miyazaki, Japan

^3^ Department of Hemovascular Medicine and Artificial Organs, Faculty of Medicine, University of Miyazaki, Miyazaki, Japan

**S-Table 1. Characteristics of the three analysed cohorts**

| Cohort | n=1186 | n=1174 | n=920 | p-value |
| --- | --- | --- | --- | --- |
| Age, years | 67.5 (12.7) | 67.5 (12.6) | 67.8 (12.6) | 0.82 |
| Gender, women, % | 42.8 | 42.8 | 43.5 | 0.95 |
| NT-proBNP, pg/mL | 4308 (2055-10800) | 4290 (2040-10796) | 4455 (2150-10383) | 0.96 |
| Log NT-proBNP, pg/mL | 3.70 (0.53) | 3.69 (0.53) | 3.70 (0.51) | 1.00 |
| Dry weight, kg |  |  |  |  |
| Women | 46.9 (9.5) ^a^ | 46.9 (9.5) | 46.7 (9.4) | 0.96 |
| Men | 57.4 (10.1) ^b^ | 57.4 (10.1) | 57.6 (10.0) | 0.95 |
| Inter-dialytic body weight gain, % | 4.67 (1.80) | 4.678 (1.80) | 4.64 (1.84) | 0.90 |
| Log dialysis vintage, months | 1.90 (0.36) | 1.90 (0.36) | 1.92 (0.36) | 0.46 |
| CVD history, % | 31.1 | 31.3 | 31.8 | 0.94 |
| Basal kidney disease, diabetes, % | 24.4 | 24.5 | 23.2 | 0.73 |

NT-proBNP, median (25-75 percentile)

The per cent of inter-dialytic body weight gain was calculated as mean 3-consecutive inter-dialytic body weight gain (kg) comparable to dry weight (kg)

Missing number; a, n=5; b, n=7

**S-Table 2. Number of deaths within two follow-up periods**

|  | Up to 3.5-year follow-up | | | | Up to 7-year follow-up | | | |
| --- | --- | --- | --- | --- | --- | --- | --- | --- |
|  | Group-Y | Group-O | Total | *P*-value | Group-Y | Group-O | Total | *P*-value |
| All-cause death | 55 | 106 | 161 | <0.01 | 125 | 181 | 306 | <0.01 |
| Non-malignancy-related death | 46 | 98 | 144 | <0.01 | 106 | 168 | 274 | <0.01 |
| CVD-related death | 28 | 35 | 63 | <0.01 | 62 | 65 | 127 | <0.01 |

CVD, cardiovascular disease

**S-Table 3. Main causes of death within two follow-up periods**

|  | Causes of death | 3.5-year follow-up | 7-year follow-up |
| --- | --- | --- | --- |
| Non-malignancy-related death | CVD-related death |  |  |
|  | CHF | 29 | 49 |
|  | sudden death | 10 | 28 |
|  | haemorrhagic stroke | 9 | 17 |
|  | ischaemic stroke | 4 | 9 |
|  | acute myocardial infarction | 6 | 13 |
|  | aortic dissection or aortic rupture | 2 | 3 |
|  | pneumonia | 22 | 36 |
|  | sepsis | 12 | 22 |
|  | dialysis withdrawal or senile deterioration | 19 | 33 |
|  | others or unknown | 31 | 64 |
| Malignancy-related death |  | 17 | 32 |

CHF, congestive heart failure; CVD, cardiovascular disease

**S-Table 4. Risks of basic confounders for all-cause mortality**

|  | Total | | Group-Y | | Group-O | |
| --- | --- | --- | --- | --- | --- | --- |
|  | HR (95% CI) | p-value | HR (95% CI) | p-value | HR (95% CI) | p-value |
| Age, +1SD year | 1.85 (1.55 to 2.22) | <0.01 | 1.58 (1.12 to 2.23) | 0.01 | 2.27 (1.49 to 3.46) | <0.01 |
| Sex, men | 1.12 (0.85 to 1.49) | 0.42 | 1.32 (0.86 to 2.00) | 0.20 | 0.92 (0.62 to 1.38) | 0.70 |
| DW standard score, +1SD | 0.71 (0.60 to 0.84) | <0.01 | 0.66 (0.53 to 0.84) | <0.01 | 0.74 (0.58 to 0.95) | 0.02 |
| Alb, +1SD g/dL | 0.78 (0.66 to 0.92) | <0.01 | 0.80 (0.63 to 1.02) | 0.08 | 0.74 (0.58 to 0.93) | 0.01 |
| Log CRP, +1 | 1.48 (1.17 to 1.86) | <0.01 | 1.54 (1.09 to 2.17) | 0.01 | 1.47 (1.06 to 2.04) | 0.02 |
| SBP, +1SD mmHg | 1.11 (0.97 to 1.27) | 0.12 | 1.21 (0.97 to 1.51) | 0.09 | 1.03 (0.86 to 1.23) | 0.74 |
| non-HDL, +1SD mg/dL | 1.04 (0.91 to 1.19) | 0.56 | 1.07 (0.88 to 1.30) | 0.49 | 1.02 (0.84 to 1.24) | 0.83 |
| Log HD vintage, +1 | 0.70 (0.47 to 1.03) | 0.07 | 0.59 (0.32 to 1.10) | 0.10 | 0.98 (0.58 to 1.67) | 0.94 |
| CTR, +1SD % | 1.22 (1.05 to 1.41) | 0.01 | 1.23 (0.98 to 1.53) | 0.08 | 1.21 (0.99 to 1.48) | 0.06 |
| Basal kidney disease (diabetes) | 1.28 (0.96 to 1.72) | 0.10 | 1.41 (0.89 to 2.24) | 0.15 | 1.03 (0.67 to 1.59) | 0.89 |
| IDBG, +1% | 0.98 (0.92 to 1.05) | 0.57 | 1.03 (0.93 to 1.14) | 0.63 | 0.94 (0.86 to 1.04) | 0.23 |
| Current smoking | 1.26 (0.86 to 1.85) | 0.23 | 1.05 (0.64 to 1.73) | 0.85 | 1.35 (0.73 to 2.52) | 0.34 |
| Antihypertensive use | 0.83 (0.61 to 1.12) | 0.23 | 1.03 (0.63 to 1.71) | 0.90 | 0.74 (0.50 to 1.11) | 0.14 |
| Past CVD history | 1.44 (1.11 to 1.86) | 0.01 | 1.42 (0.95 to 2.12) | 0.09 | 1.51 (1.06 to 2.15) | 0.02 |

Alb, albumin; CRP, C-reactive protein; CVD, cardiovascular disease; CTR, cardiothoracic ratio; DW, dry weight; DW, dry weight; HDL, high density lipoprotein; HD, haemodialysis; IDBG, inter-dialytic body weight gain; SD, standard deviation; SBP, systolic blood pressure

**S-Table 5. Hazard ratios for all-cause mortality according to the interaction terms for age and log NT-proBNP**

|  |  | β | HR (95% CI) | p-value |
| --- | --- | --- | --- | --- |
| Without interaction | |  |  |  |
|  | Age, +1 years | 0.07 | 1.07 (1.06 to 1.09) | <0.01 |
|  | Log NT-proBNP, +1 | 1.08 | 2.93 (2.35 to 3.67) | <0.01 |
| With interaction | |  |  |  |
|  | Age, +1 years | 0.18 | 1.20 (1.10 to 1.31) | <0.01 |
|  | Log NT-proBNP, +1 | 3.22 | 25.02 (4.89 to 127.98) | <0.01 |
|  | Age×Log NT-proBNP product | -0.03 | 0.97 (0.95 to 0.99) | 0.01 |
|  |  |  |  |  |
|  |  | β | HR (95% CI) | p-value |
| Without interaction | |  |  |  |
|  | Age, +1 years | 0.05 | 1.05 (1.04 to 1.07) | <0.01 |
|  | Log NT-proBNP, +1 | 0.73 | 2.08 (1.57 to 2.77) | <0.01 |
| With interaction | |  |  |  |
|  | Age, +1 years | 0.09 | 1.10 (1.01 to 1.20) | 0.04 |
|  | Log NT-proBNP, +1 | 1.53 | 4.61 (0.92 to 23.15) | 0.06 |
|  | Age×Log NT-proBNP product | -0.01 | 0.99 (0.97 to 1.01) | 0.33 |

Results of Cox analysis of upper panel is unadjusted model, whereas, lower panel is fully adjusted with DW standard score (+1SD), sex, Alb (+1SD), log CRP, CVD history, SBP (+1SD), CTR (+1SD), non-HDL-c (+1SD), log dialysis vintage, current smoking, inter-dialytic body weight gain comparable to dry weight (+1%), basal kidney disease (diabetes), and antihypertensive medicine use

**S-Table 6. Risks of tertiles log NT-proBNP for mortality up to 3.5-year follow-up**

|  |  |  | All-cause death | | Non-malignancy-related death | | CVD-related death | |
| --- | --- | --- | --- | --- | --- | --- | --- | --- |
|  |  |  | HR (95% CI) | p-value | HR (95% CI) | p-value | HR (95% CI) | p-value |
| Unadjusted | Total cohort | T1 | ref |  | ref |  | ref |  |
|  |  | T2 | 3.67 (2.03 to 6.65) | <0.01 | 4.29 (2.22 to 8.30) | <0.01 | 10.95 (2.57 to 46.69) | <0.01 |
|  |  | T3 | 8.65 (4.94 to 15.14) | <0.01 | 9.86 (5.27 to 18.47) | <0.01 | 24.47 (5.91 to 101.27) | <0.01 |
|  | Group-Y | T1 | ref |  | ref |  | ref |  |
|  |  | T2 | 3.51 (1.16 to 10.66) | 0.03 | 12.01 (1.56 to 92.38) | 0.02 | n/a |  |
|  |  | T3 | 10.44 (3.72 to 29.28) | <0.01 | 37.20 (5.09 to 272.02) | <0.01 | n/a |  |
|  | Group-O | T1 | ref |  | ref |  | ref |  |
|  |  | T2 | 1.63 (0.95 to 2.80) | 0.08 | 1.44 (0.81 to 2.55) | 0.21 | 2.39 (0.91 to 6.30) | 0.08 |
|  |  | T3 | 3.19 (1.93 to 5.25) | <0.01 | 3.21 (1.92 to 5.35) | <0.01 | 3.51 (1.37 to 8.98) | 0.01 |
| Adjusted | Total cohort | T1 | ref |  | ref |  | ref |  |
|  |  | T2 | 2.31 (1.27 to 4.21) | 0.01 | 2.63 (1.35 to 5.10) | <0.01 | 7.87 (1.83 to 33.82) | 0.01 |
|  |  | T3 | 3.53 (1.98 to 6.29) | <0.01 | 3.71 (1.94 to 7.08) | <0.01 | 11.95 (2.83 to 50.51) | <0.01 |
|  | Group-Y | T1 | ref |  | ref |  | ref |  |
|  |  | T2 | 2.86 (0.93 to 8.82) | 0.07 | 9.79 (1.26 to 75.96) | 0.03 | n/a |  |
|  |  | T3 | 5.60 (1.94 to 16.13) | <0.01 | 19.21 (2.58 to 142.95) | <0.01 | n/a |  |
|  | Group-O | T1 | ref |  | ref |  | ref |  |
|  |  | T2 | 1.33 (0.77 to 2.31) | 0.31 | 1.16 (0.65 to 2.06) | 0.63 | 2.05 (0.77 to 5.46) | 0.15 |
|  |  | T3 | 1.78 (1.05 to 3.04) | 0.03 | 1.70 (0.98 to 2.94) | 0.06 | 2.03 (0.76 to 5.45) | 0.16 |
|  |  |  |  |  |  |  |  |  |

Adjusted model. Adjusted with age (+1SD), DW standard score (+1SD), sex, Alb (+1SD), log CRP, CVD history, SBP (+1SD), CTR (+1SD), non-HDL-c (+1SD), log dialysis vintage, current smoking, inter-dialytic body weight gain comparable to dry weight (+1%), basal kidney disease (diabetes), and antihypertensive medicine use

Alb, albumin; CI, confidence interval; CRP, C-reactive protein; CTR, cardiothoracic ratio; DW, dry weight; HDL, high density lipoprotein; HR, hazard ratio; SBP, systolic blood pressure


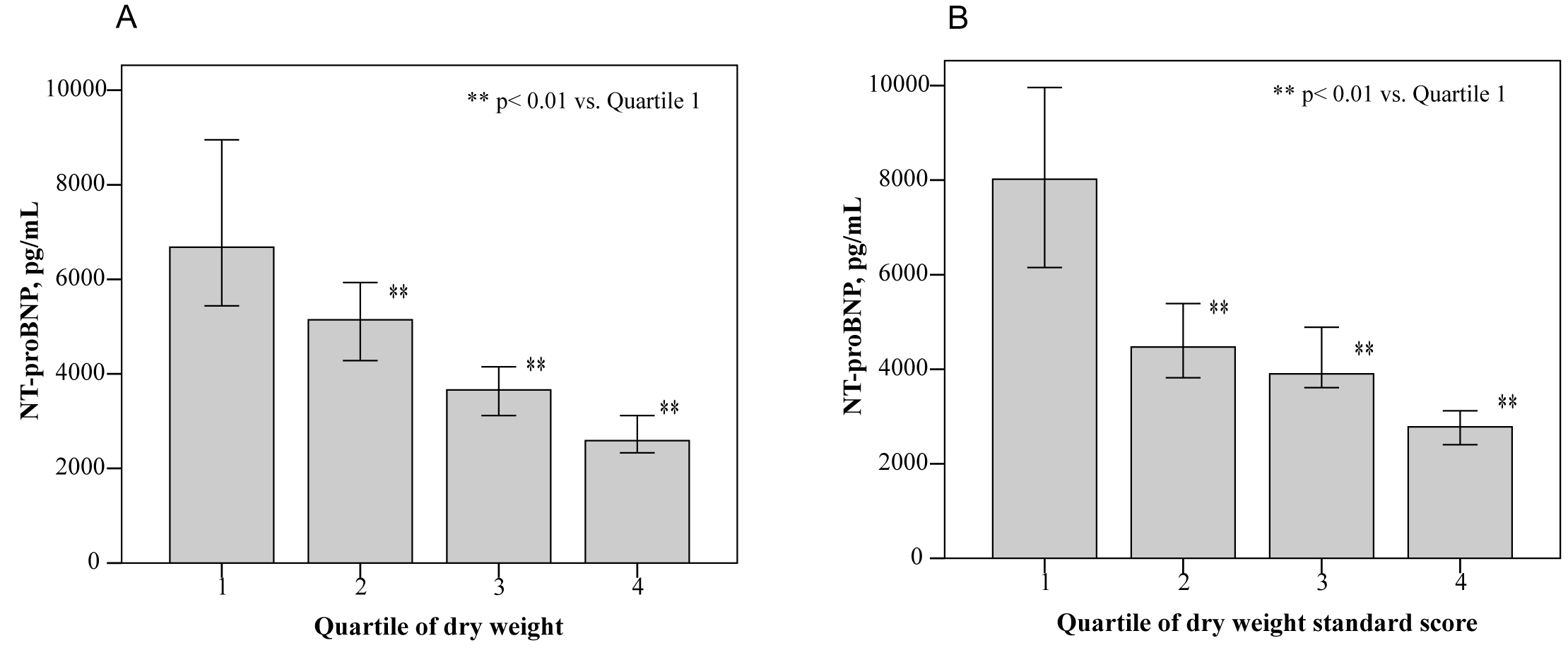


**S-Figure 1. NT-proBNP association with dry weight**

Patients were divided into quartile of raw dry weight (DW) data (A) and standard score data (B). Because DW was lower in women than in men, the DW standard score for each sex was calculated and compiled for analysis. Regardless of each quartile, NT-proBNP of quartile 1 was significantly higher than those of other quartiles. Data are expressed as median +/- 95% confidence interval. P-values were checked by nonparametric test.


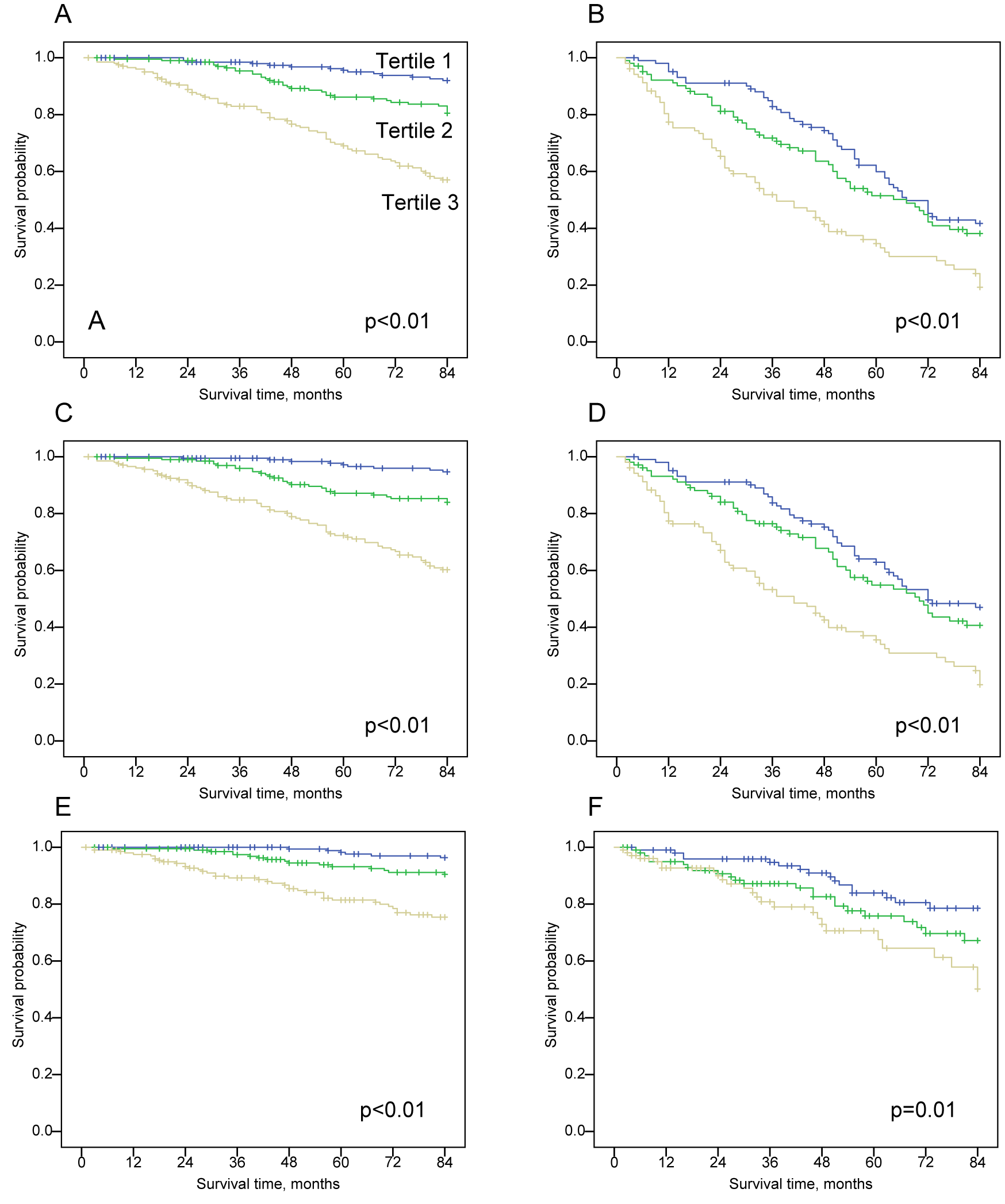


**S-Figure 2. Kaplan–Meier survival estimate curves for all-cause mortality**

Kaplan-Meier survival estimate curves showed that tertiles of log NT-pro BNP were significantly and clearly differentiated for all-cause mortality in Group-Y (A) and Group-O (B), non-malignancy-related mortality in Group-Y (C) and Group-O (D), as well as for CVD-related mortality (E, F). All Kaplan-Meier estimate curves are statistically significant based on log-rank test; *p*-values are shown. However, patients in Group-O (B, D, and F) reached outcome earlier than those in Group-Y (A, C, and E).
